# Supplementary material for: Dynamical mean-field theory for a highly heterogeneous neural population with graded persistent activity of the entorhinal cortex
Source: PLoS Comput Biol. 2025 Sep 16;21(9):e1013484. doi: 10.1371/journal.pcbi.1013484 (PMC12456838; doi:10.1371/journal.pcbi.1013484)
Supplement: S1 Appendix — (PDF) [file pcbi.1013484.s001.pdf]

Appendix

Continuous distribution of heterogeneity in intrinsic decay rates

In the main text, to clarify the role of intrinsic heterogeneity in decay rates on population dynamics, we have presented network dynamics in which the intrinsic decay rates of individual neurons follow a two-valued discrete distribution. However, in the brain, neuronal heterogeneity does not always follow discrete distributions. Rather, it often follows continuous ones. For example, ramping cells and time context cells in the entorhinal cortex and hippocampus, which are considered crucial for temporal signal processing, exhibit a wide range of intrinsic time scales that are thought to follow a broad and continuous distribution across neurons [1, 2]. Here, to demonstrate that the theory developed in this paper is also applicable to broad and continuous distributions of intrinsic heterogeneity in decay rates, we study the dynamics of networks in which the decay rate γ and the feedback strength β of each neuron follow continuous, rather than two-valued, distributions.

First, to see how neurons with different parameters behave differently within the network, we selected subsets of neurons with different ranges of γ values and analyzed them separately (Fig S1). In Panel (a), we show the activity of neurons with small and large decay rates, corresponding to slower and faster time scales, respectively. The corresponding autocorrelation functions are shown in Panel (b). Panels (c) and (d) present the average autocorrelation functions within each group, along with the distributions of relaxation times computed from the autocorrelation functions of individual neurons before averaging. These results indicate variation in temporal dynamics depending on the decay rate.

Then, we numerically measured the maximum power spectrum of the network dynamics to examine how continuous heterogeneity shifts the transition point, and whether this shift is still accurately predicted by the developed theory. Fig S2 shows the same type of spectrum as in Figs (4) and (7) in the main text, but for networks in which the decay rate γ (left panel) or the feedback strength β (right panel) follows continuous distributions. As indicated by the red lines, the theoretical prediction closely matches the transition points of these networks with continuous heterogeneity.

Temporal context cell-like activity in the heterogeneous network

Here, we consider a heterogeneous recurrent network in which intrinsic time scales, in particular the feedback strengths β_i, follow continuous distributions. We numerically demonstrate that neurons in such a network exhibit response patterns similar to those of 'temporal context cells' recently observed in the entorhinal cortex [1].

Temporal context cells are a class of neurons recently identified in the entorhinal cortex. In contrast to hippocampal 'time cells', which exhibit peak activity at specific delays after a stimulus such as entering a new environment, temporal context cells respond rapidly following stimulus onset and then gradually return to baseline activity with a wide variety of decay rates. Since the elapsed time from stimulus onset can be decoded precisely from the population activity of temporal context cells, they are thought to encode temporal information that works in concert with hippocampal time cells.

To examine whether similar response patterns, specifically a rapid increase in activity followed by a slow decay with varying time constants, can be reproduced by our heterogeneous network, we applied a transient impulse input to the network and measured the resulting activity amplitudes of individual neurons. Fig S3 shows the results. For clarity, we randomly selected 30 neurons from the network and plotted their activities in ascending order of feedback strength β_i. The figure clearly shows that most

neurons exhibit a sharp increase in activity upon stimulus presentation, followed by a gradual return to baseline with variable, and often fairly long, time scales. This behavior closely resembles that of temporal context cells observed experimentally.

Memory capacity for heterogeneous networks

To assess how heterogeneity affects the computational capability of the network, we numerically evaluated the Memory Capacity (MC), which is a standard metric used to quantify the capacity of short-term memory in the reservoir computing framework [3]. MC is defined as the sum of the correlations between the past input $u(t-\tau)$ and its estimate $\hat{u}(t-\tau) = \mathbf{w}_\tau^T \mathbf{x}(t)$, where $\mathbf{x}(t)$ is the current state of the network, Specifically, $MC = \int_0^\infty MC(\tau) d\tau$, where

$$MC(\tau) = \frac{\mathrm{cov}(u(t-\tau), \hat{u}(t-\tau))^2}{\mathrm{var}(u(t-\tau))\mathrm{var}(\hat{u}(t-\tau))}$$

Here, the input $u(t)$ is an i.i.d. random sequence, and the readout weights $\mathbf{w}_\tau$ for each delay τ are determined via ridge regression to minimize the mean squared error.

Fig S4 shows the memory capacity of the heterogeneous network for various levels of heterogeneity. The memory capacity is maximized when the coupling strength is near, though slightly below, the critical value indicated by the vertical dashed lines. This result is consistent with well-known findings in reservoir computing, which suggest that computational capability is often maximized in the region known as the edge of chaos.

References

1. Bright IM, Meister MLR, Cruzado NA, Tiganj Z, Buffalo EA, Howard MW. A temporal record of the past with a spectrum of time constants in the monkey entorhinal cortex. Proc Natl Acad Sci U S A. 2020;117(33):20274–20283.

2. Tsao A, Sugar J, Lu L, Wang C, Knierim JJ, Moser MB, et al. Integrating time from experience in the lateral entorhinal cortex. Nature. 2018;561(7721):57–62.

3. Jaeger H. The “echo state” approach to analysing and training recurrent neural networks-with an erratum note. Bonn, Germany: German National Research Center for Information Technology GMD Technical Report. 2001;148(34):13.
