# Supplementary figures and images for: Dynamical mean-field theory for a highly heterogeneous neural population with graded persistent activity of the entorhinal cortex
Source: PLoS Comput Biol. 2025 Sep 16;21(9):e1013484. doi: 10.1371/journal.pcbi.1013484 (PMC12456838; doi:10.1371/journal.pcbi.1013484)

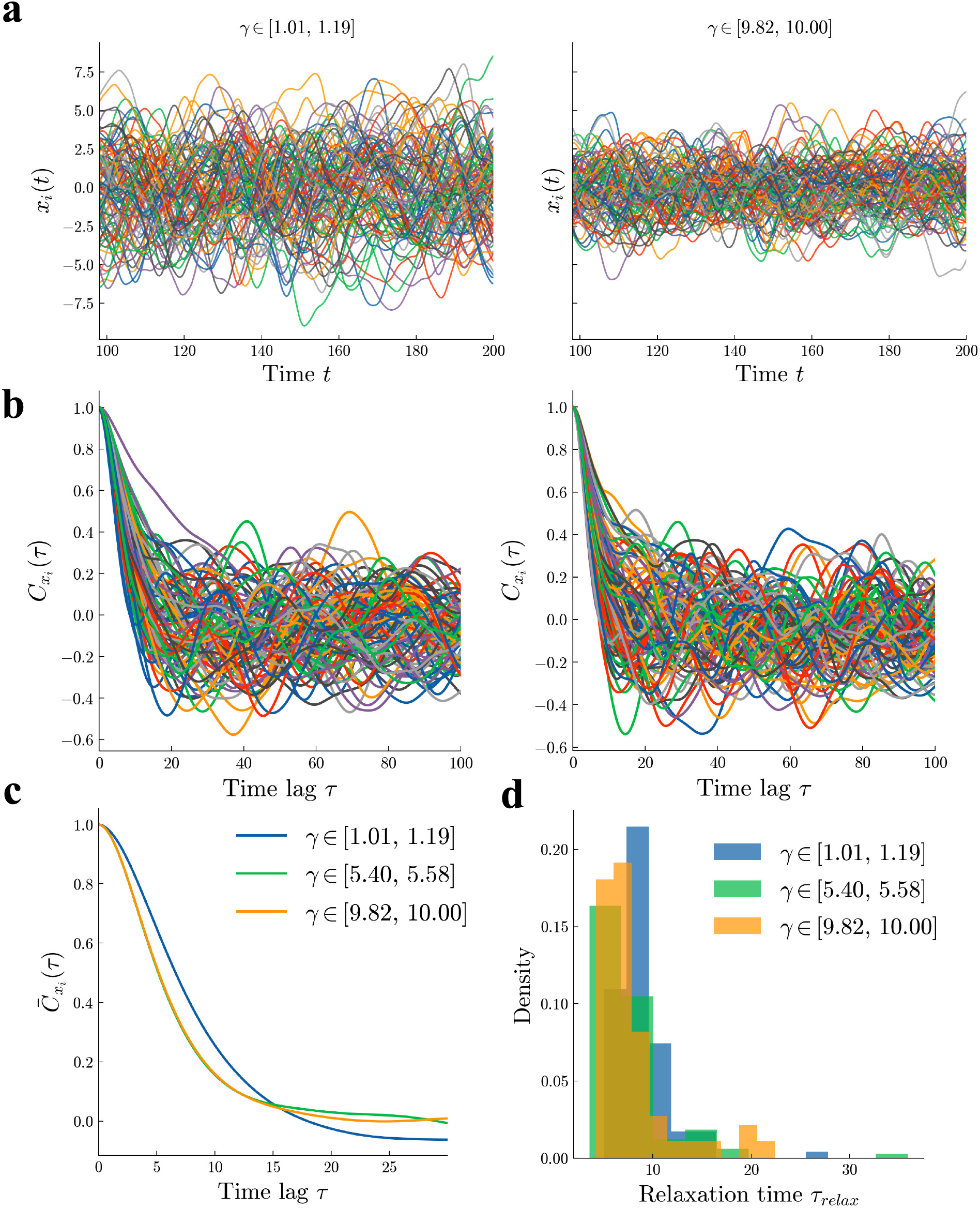

Supplement: S1 Fig — (a) Temporal profiles of the activity of neurons with small (left) and large (right) decay rate parameters, corresponding to slower and faster time scales, respectively. (b) Autocorrelation functions of the same neuron groups are shown in (a). (c) Average autocorrelation functions within subgroups of neurons with ranges of decay rate parameters. (d) Distributions of relaxation times computed from the autocorrelation functions of individual neurons prior to averaging. Decay rates γi follow a uniform distribution U[1,10] across the network. Parameters N = 5000 and g = 2.0 are used. (TIF) [file pcbi.1013484.s002.tif]

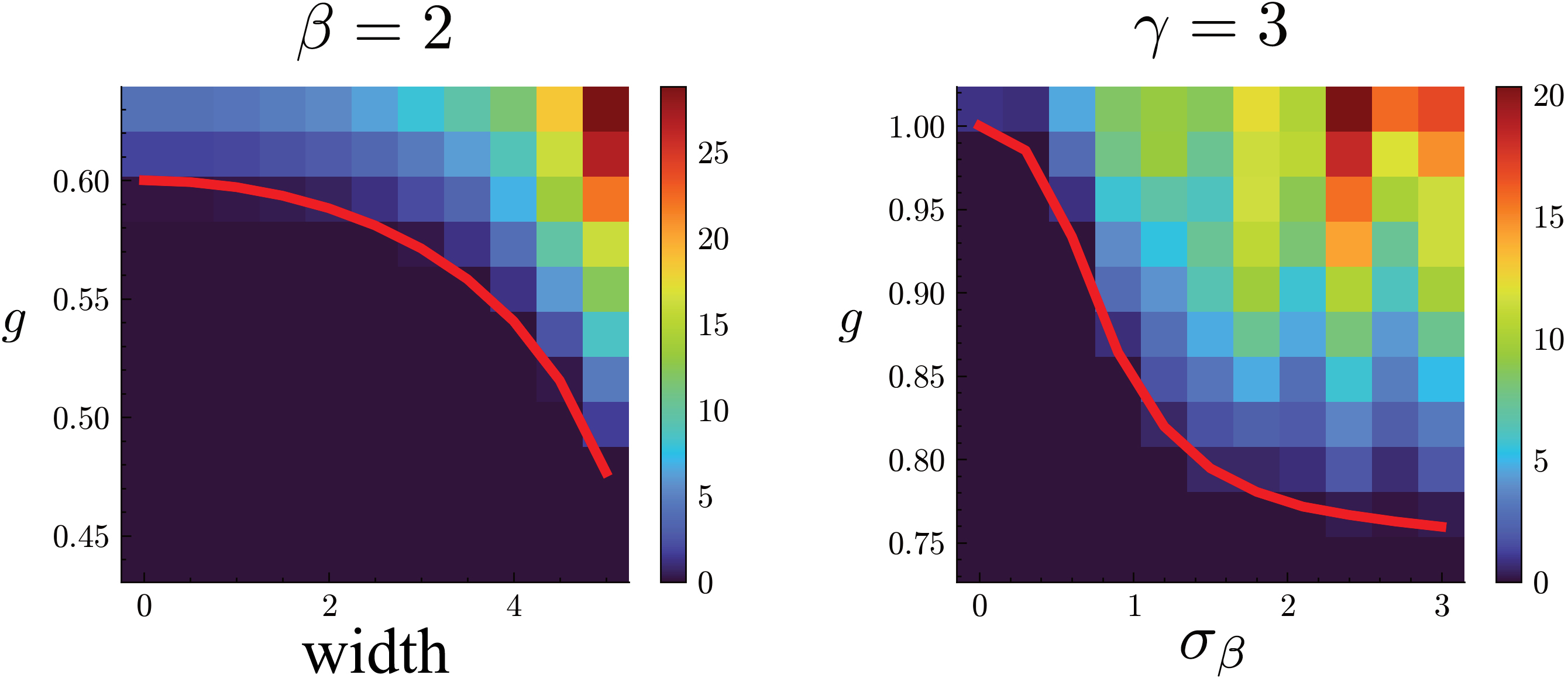

Supplement: S2 Fig — The left panel shows the result for a network in which the decay rates γi follow a uniform distribution which center is 5. The right panel shows the result for a network in which the feedback strengths βi follow a truncated normal distribution with mean 0 and range [–2,2]. Red lines indicate the theoretical predictions. (TIF) [file pcbi.1013484.s003.tif]

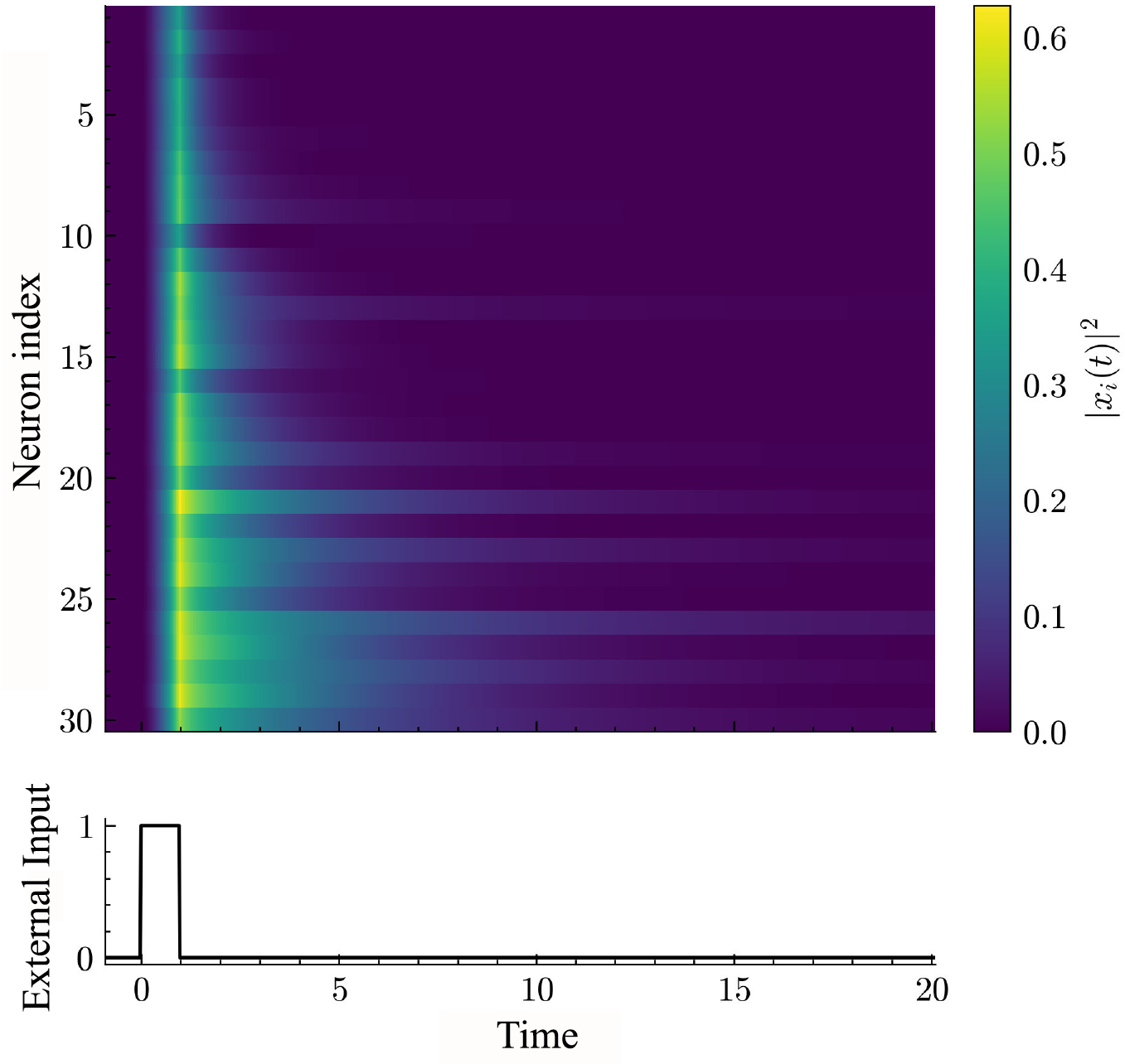

Supplement: S3 Fig — The upper heatmap shows the response amplitudes of individual neurons to the same pulse input applied to the network. Neurons are sorted from top to bottom in ascending order of their feedback strength βi. The lower panel shows the external input, which is shared by all neurons. The network operates in a stable regime where g = 0.15 < gc = 0.183, and the feedback strengths βi follow a uniform distribution U[0,2.9]. Other parameters are N = 3000 and γ=3.0. (TIF) [file pcbi.1013484.s004.tif]

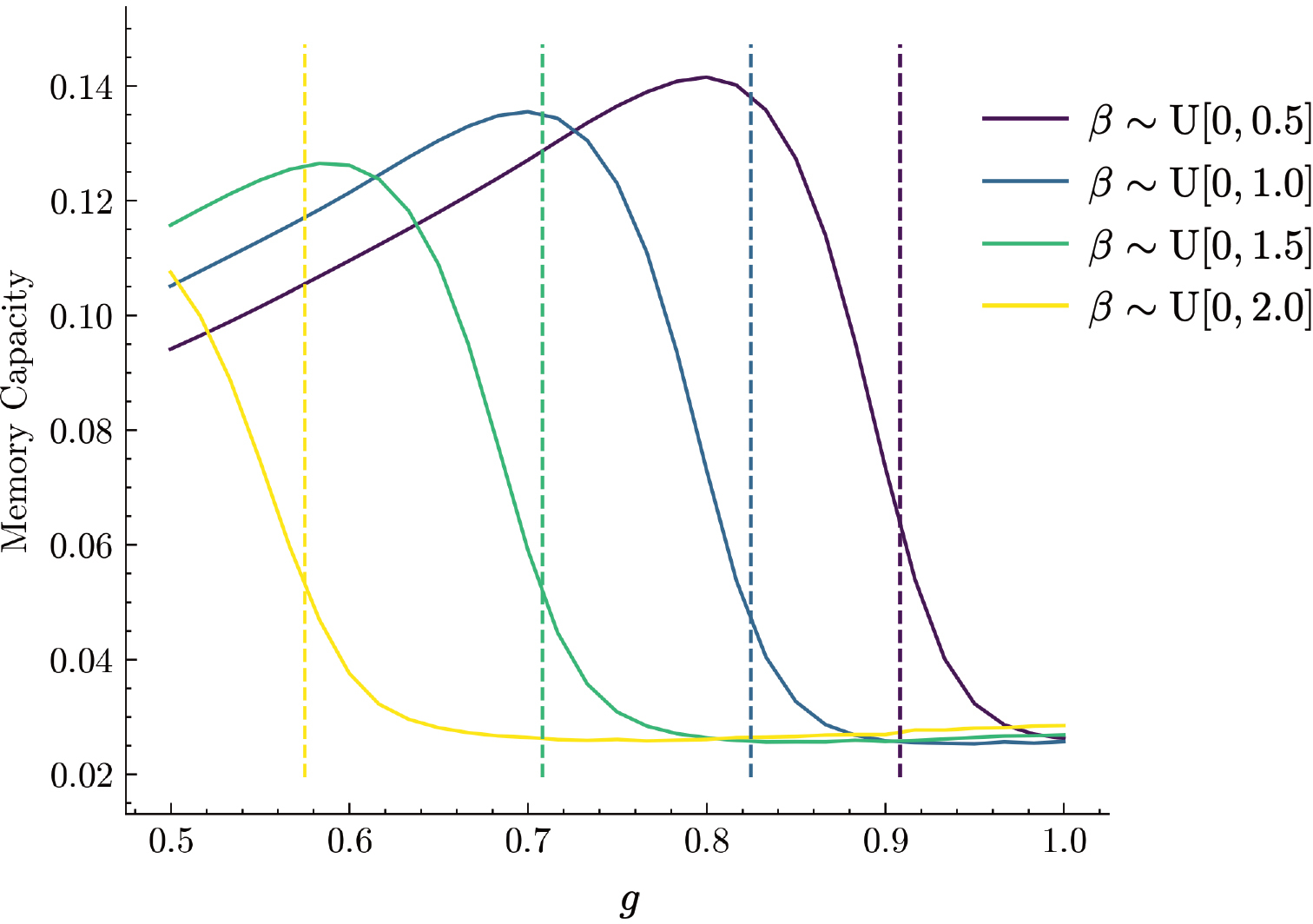

Supplement: S4 Fig — Vertical dashed lines indicate the critical coupling strengths for each network. The input signal u(t) is modeled as Gaussian white noise, and the time evolution of the network is computed using the Euler–Maruyama method for stochastic differential equations. The other parameters are N = 3000, γ=3, u(t)∼𝒩(0,σin2dt), σin=0.1, and dt = 0.02. (TIF) [file pcbi.1013484.s005.tif]
