# Supplementary material for: Dynamical mean-field theory for a highly heterogeneous neural population with graded persistent activity of the entorhinal cortex
Source: PLoS Comput Biol. 2025 Sep 16;21(9):e1013484. doi: 10.1371/journal.pcbi.1013484 (PMC12456838; doi:10.1371/journal.pcbi.1013484)
Supplement: Attachment — Submitted filename: reply_tomita_20250809.pdf [file pcbi.1013484.s006.pdf]

Dear editor

We sincerely thank you and the reviewers for your valuable comments and the positive evaluation of our manuscript. We have carefully revised the manuscript and conducted additional numerical calculations (Figures S1, S2, S3 and S4 in the Supporting Information) to address all the concerns raised by the reviewers. We believe that the revision, along with the additional numerical results, provides substantial reinforcement to the hypothesis and conclusions of our study.
Below, we provide a point-by-point response to each comment.

Additional Editor Comments :
The authors should consider simulating a wider range of time scales, clarifying the mean field equations, and exploring the use of chaotic dynamics to encode temporal information.

Thank you for the constructive comment. We have addressed all of the points raised. Please see our detailed responses in the following replies to the reviewers.

Reviewer #1:
This paper presents an analytical method based on dynamical mean field theory and Fourier transform, and applies it to study the transition to chaos for randomly-connected neural networks where neurons have prolonged firing due to self-feedback mechanism. The authors show that when the network consists of two populations of neurons with long and short timescales for the self-feedback mechanism, the transition to chaos happens earlier (at lower coupling strength g). In addition, using the same method, the authors show that when the self-feedback timescales are sampled from a Gaussian distribution, the transition to chaos occurs later with higher values of sigma (the standard deviation of the Gaussian distribution), contrary to a previous result.
The technical aspect of the work is impressive, and the results seem important and relevant for researchers interested in how the type of slow neural activity observed in many parts of the brain could be beneficial for memory functions. On the other

hand, I do think there are a few places where the paper could be made accessible for a wider audience:

We sincerely thank the reviewer for the positive evaluation of the manuscript and for the constructive suggestions for its improvement. We have revised the manuscript to address all of the comments and have performed additional numerical computations. Below, we provide our point-by-point responses.

1. The author mentioned briefly that chaotic activity (and therefore a larger fraction of slow neurons) could be useful in encoding temporal information. I think it would make the paper be of interest a wider audience if the authors could show this using an example. There have been proposals of how ramping cell and time cells can be used to encode temporal information (e.g. Shankar and Howard, Neural Computation 2002). There have been other proposals for network mechanisms that benefit temporal information encoding, for example using non-normal dynamics (e.g. Ganguli, Huh and Sompolinsky, PNAS 2008; Goldman, Neuron 2009). Both classes of models do not have chaotic activity. Rather they use the transient activity to encode temporal information before going back to the resting state. So I think it would be useful for readers to see how an alternative mechanism using chaos could also benefit temporal information processing.

We sincerely thank you for pointing out important issues, and we apologize for the confusing description in the previous version of the manuscript. Our earlier explanation of the chaotic regime lacked clarity. What we meant to express is that heterogeneity expands the chaotic dynamical regime, which in turn modulates the region near the transition between the stationary and dynamical regimes. This modulation, especially around the so-called edge of chaos, is functionally important because it affects the network's capability for temporal information processing. To clarify this point and to demonstrate that heterogeneity indeed modulates the temporal coding capability, we have revised two paragraphs beginning at line 342 and conducted additional numerical simulations to evaluate the memory capacity of the heterogeneous network (Figure S4). Memory capacity

is a quantitative measure of how long temporal signals can be stored by and decoded from the network. The simulation results clearly show that memory capacity is maximized near the transition boundary, specifically just below the critical point, in agreement with previous literature. This finding supports the idea that intrinsic heterogeneity can effectively modulate the temporal coding ability. Please see the revised Supplementary Information and Figure S4 for further details.
Regarding the literature you kindly provided, we once again thank you for drawing our attention to these important studies. In particular, while we were not previously aware of it, we now recognize that the work by Shankar and Howard, along with their more recent follow-up study, is closely related to our findings. To incorporate this connection into our discussion, we have added two new paragraphs in the Discussion section (lines 359–381 and lines 382–393), where we discuss the relationship between our work and theirs. We have also found that our model reproduces response dynamics resembling those of 'temporal context cells,' which are closely related to the work of Shankar and Howard and were identified by one of the authors. Please see the newly added sentences (lines 408–413) in the main text, Supporting Information, and Figure S3 for details. We also added a reference to the work of 'temporal context cells' in lines 12-15 of Introduction.

2. I am not an expert of dynamic mean-field theory. While the authors did a good job stepping through the calculation, there are still a few places where I could not follow, and I believe it would benefit average audience if the authors could provide a bit more hand-holding in the calculation:
a. Equation 22: is P(¥ksi^{¥alpha}_{m, i}) the probability of x _{m, i} and a_{m, i}, given a particular realization of the noise ¥ksi? If so, I think it might be helpful to point this out explicitly or write it as P(x, a | ¥ksi)

We apologize for the insufficient explanation in the previous version. The distribution $P(¥xi^{¥alpha}_{m, i})$ is Gaussian, and it appears here through a variable transformation implied by equation (21), followed by marginalization

over a Gaussian variable. To clarify this point, we have added a new equation ((22) in the current manuscript, so the former equation (22) is now numbered (23)) along with a clarifying sentence preceding it.

b. Equation 22, second line: why is there an "i" in the "2*¥pi*i" in the denominator? I didn't remember the "i" from the Fourier expression of the delta function.

Thank you for addressing this point. The imaginary unit appears here because, following the standard approach in DMFT, we have defined k as an imaginary variable. To make this explicit, we have added the range of integration to the equation.

c. Equation 24: I didn't get how the moment generating function Z can be transformed to the form k^2*¥sigma*dt

Thank you for the comment. This follows from the fact that the moment generating function of a Gaussian distribution is given by the exponential of a quadratic expression; that is, $Z(t) ¥equiv E[e^{tX}] = ¥exp(¥mu t + ¥sigma^2 t^2 / 2)$ when X follows $N(¥mu, ¥sigma^2)$. We have added a sentence below equation (25) to briefly explain this.

d. Equation 35: why the signs in the exponential (+ ¥psi * Q2 * ¥psi – N/g^2 * Q1 * Q2) are reversed compared to equation 34?

We sincerely apologize for the confusion. This was a typographical error. We have corrected the sign and confirmed that this modification does not affect any of the subsequent results.

e. Equation 39: I could not follow how are the extrema derived. It would be helpful to show the intermediate steps.

Thank you for the comment. In order to clarify the derivation of the extrema, we have introduced the action S in equation (38) in the current manuscript, slightly rewrite sentences before (39) and added two equations below equation (39) to explicitly show the form of the saddle-point equations in the current context. The extrema (40) are obtained by directly solving these equations.

f. Text below Equation 40: why can we conclude from Equation 40 that each neuron is driven by a Gaussian process with mean 0 and autocorrelation C_{¥psi}?

Thank you for pointing this out. This result is obtained because the expression of the moment generating functional is exactly the same as that of the moment generating functional without coupling and with noise $¥sigma_x = g^2 C_{¥phi}$. To clarify this, we have revised the sentences following equation (41) in the current manuscript. In addition, to avoid possible confusion, we have explicitly introduced the suffix $¥alpha$ to denote the noise strength $¥sigma_¥alpha$ in equation (25, 26).

I want to emphasize that many of these questions may just be due to my lack of familiarity with DMFT, so the authors may choose to just respond here without changing the manuscript. But I believe this gives an idea of where an average reader might find difficult to follow. Based on this the authors may want to consider providing a bit more details and explanations for some parts of the mathematical derivation.

Thank you for your kind consideration and for carefully reviewing the details of our theoretical development. We believe that your suggestions regarding the theoretical derivation have significantly improved the overall readability of the manuscript and enhanced its accessibility for a broader audience beyond experts in DMFT.

3. The distribution of the eigenvalues shown in Figure 5 is interesting. There seems to be a transition from 2 bulks to 3 bulks when p is increased. It would be

interesting to study this a bit more. For example, do the eigenmodes in different bulks consist of neurons with different timescales? Is there a corresponding transition in the distribution of single-cell timescales (e.g. as calculated by the decay time constant of its autocorrelation) from a bi-modal distribution to a tri-modal one?

Thank you very much for your constructive suggestions. In fact, after the initial submission of this manuscript, we have successfully developed a new theoretical framework to analyze the intriguing eigenvalue distribution observed in our system. This new theory enables us to analytically elucidate how the bulk of the eigenvalue distribution is governed by the heterogeneity in the system parameters. However, due to space limitations and the fact that this development goes beyond the intended scope of the current manuscript, we have decided not to include a detailed exposition here. If permitted, we would like to present and discuss this new theoretical framework in a separate future paper.

4. Line 351-352: the author mentioned that future work could look at the relationship between the dynamics of the network they studied, and the characteristic activity patterns observed in the entorhinal cortex. I think even for the current setup, there are something that could be examined. For example, are there single units in the network that show ramping activity or time-cell-like activity (localized temporal receptive field)? In addition, it is known that the time constants of time cells and ramping cells span a continuum. Do the timescales of single cell response in this network also form a continuum (this might relate to the eigenvalue distribution mentioned in point #3)?

Thank you so much for the insightful and kind suggestion. Since the scope of our theory is not limited to a two-valued distribution of time constants, we put a comment to this at lines 138-141 and extended our analysis to the case of a continuous distribution of decay rates and examined how differences in this parameter influence the temporal profiles of neurons in the network (Figures S1 and S2; see Supplemental Information for details). Furthermore, we found that

the amplitude of single-unit activity in the network exhibits a temporal profile closely resembling that of the recently identified "temporal context cells" in the entorhinal cortex (Figure S3). In particular, the response shows a sharp increase in amplitude at stimulus onset, followed by a gradual return to baseline with long, variable, and continuously distributed time scales. We believe that the inclusion of these new results has significantly strengthened the overall impact and clarity of the paper. Once again, we are deeply grateful for your thoughtful and constructive comment.

Minor points
1. Fig 6: it would be more helpful for readers if legend was included in the figure.
2. Line 336-337: "external inputs do not decay" – I think the more accurate expression is "network activity triggered by external inputs do not decay"
3. Line 497: excepting : except

We appreciate your careful review. We have addressed all of your comments accordingly.

Reviewer #2: Present study proposes a computational method that allows one to apply dynamical mean-field theory to neural populations with different time scales. The problem is clearly articulated, and the proposed solution--averaging in Fourier space--is elegant. I find the analysis sound and emphasis on distinguishing intrinsic heterogeneity and connection heterogeneity bares important implications for future work.

We sincerely thank the reviewer for the positive assessment and thoughtful suggestions. In response, we have revised the manuscript and conducted additional numerical computations. Below, we provide the details of the revisions.

My main concern is the simplification of the model to just two time-scale levels (high vs. low). Empirical recordings from the entorhinal cortex suggest a more continuous spectrum of time scales (e.g. Tsao et al, 2018 Nature; Bright et al.,

2020, PNAS). It would be interesting to explore whether a transition point can be derived under such highly variable time scales. At minimum, a discussion of this point would be valuable. Ideally, a simulation could strengthen the papers appeal to a broader audience.

Thank you very much for the insightful and constructive suggestions. While we focused on a two value discrete distribution of time scales to clarify the role of intrinsic heterogeneity, the theory developed here is also applicable to networks with a continuous spectrum of intrinsic time scales, as you anticipated. To investigate the population dynamics of such networks and the dynamical properties of individual neurons, we put a comment at lines 138-141 in the main text and conducted additional numerical simulations using our theoretical framework.

Figures S1 and S2 present the results. Figure S1 illustrates the diversity of temporal dynamics across neurons, with different decay rates leading to distinct autocorrelation profiles. Figure S2 shows that the shift in the transition point indeed occurs even in the presence of a highly variable and continuous distribution of time scales, consistent with the theoretical prediction.

We also sincerely thank you for directing our attention to the work by Bright et al. (2020, PNAS). We added a reference to the work at lines 12-15 in Introduction. Although we were not previously aware of it, through an additional simulation (Figure S3), we have found that our model exhibits response dynamics that are remarkably similar to those of ‘temporal context cells’ reported in their study. Specifically, the response of each neuron in the network shows a sharp increase in amplitude at the onset of the stimulus, followed by a gradual return to the baseline with long, variable, and continuously distributed time scales. Please see the newly added sentences (lines 408 to 413) in the main text and the Supporting Information for further details.

Minor comments:

1. The Github link does not work.

We apologize for the oversight and thank you for letting us know. We have changed github link to https://github.com/fuuta/multiD_hetero_DMFT and corrected the issue.

2. Several parameters are discussed throughout the paper but are not always referred consistently. It would make it easier to track parameters are referenced with both parameter name and its role in the function (e.g., coupling strength g) throughout, especially when a parameter is reintroduced several pages after its initial introduction.

Thank you for the helpful comment. We have revised the manuscript throughout to ensure that both the parameter name and its role are consistently provided not only at their initial introduction but also when the parameters are reintroduced at the beginning of each section.
